# Engaging Dialysis Teams in Shared Decision-Making Conversations With Patients to Improve Rates of Kidney Transplantation

**DOI:** 10.1016/j.xkme.2026.101310

**Published:** 2026-02-27

**Authors:** Anne M. Huml, Lindsey M. Maclay, Syed Ali Husain, Miko Yu, Heedeok Han, Jesse D. Schold, Sumit Mohan

**Affiliations:** 1 Department of Kidney Medicine, Cleveland Clinic, Cleveland, OH; 2 Division of Nephrology, Department of Medicine, Vagelos College of Physicians and Surgeons, Columbia University, New York, NY; 3 Columbia University Renal Epidemiology Group, New York, NY; 4 Department of Surgery, University of Colorado, Anschutz Medical Campus, Aurora, CO; 5 Department of Epidemiology, School of Public Health, University of Colorado, Anschutz Medical Campus, Aurora, CO; 6 Department of Epidemiology, Mailman School of Public Health, Columbia University, New York, NY

**Keywords:** kidney transplant, organ offers, shared decision making, wait listing

## Abstract

**Rationale & Objective:**

Increasing access to kidney transplantation requires patients to be active on the waiting list and empowered to consider donor offers that are consistent with their preferences. We sought to develop and refine patient-centered communication tools for shared decision-making conversations within dialysis facilities about active status on the waiting list, consent for hepatitis C and high-kidney donor profile index organ offers, and living donation. Conversations may help patients improve their probability of kidney transplantation.

**Study Design:**

A qualitative study convening focus groups of stakeholders from dialysis and kidney transplantation. Iterative study design to understand practice patterns to design and refine communication tools with stakeholder input.

**Setting & Participants:**

A total of 28 stakeholders, including 13 providers, 13 patients, and 2 care partners, participated in virtual focus groups. Providers represented a diverse, national sample of physicians, advanced practice providers, technicians, nurses, and social workers.

**Analytical Approach:**

Thematic analysis of focus group transcripts using a constant comparative approach to identify themes and subthemes. Thematic coding was refined iteratively during the analytic process, and consensus coding was used to determine final themes and subthemes. A subset of the communication tools was quantitatively evaluated using a validated tool.

**Results:**

Information about kidney transplantation exchanged between patients and dialysis providers was described as transactional and passive. Successful lived experiences of other patients treated with dialysis empowered discussions while providers’ cursory knowledge of the nuances of kidney transplantation acted as barriers to shared decision-making conversations. Personalized information, tailored to the appropriate health literacy level, and delivered through multiple modalities were identified as necessary characteristics of communication tools. Conveying statistics and risks and benefits of different organ types were positive features of the communication tool, while potential implementation strategies required refinement.

**Limitations:**

Findings may not represent all practice patterns and patient preferences.

**Conclusions:**

Understanding existing practice patterns and preferred content for communication tools can be used to engage dialysis teams in shared decision-making about transplantation.

## Plain-Language Summary

Patients treated with dialysis must go through a multi-step process to be evaluated for a kidney transplant. Once they are on the kidney transplant waiting list, patients may not know whether they are on inactive versus active status and realize the potential risks and benefits of consenting to different organ offer types. In this study, we conducted focus groups with patients, providers, and care partners to (1) understand current practices for communication about transplant in dialysis facilities, and (2) obtain input about a tool that can be used for increased communication with patients about the types of donors that they have agreed to receive. The goal of this work is to improve access to kidney transplantation among waitlisted patients.

Kidney transplantation is the preferred treatment choice for patients with end-stage kidney disease (ESKD), yet only a small proportion of patients with incident ESKD are counseled about their transplant options, and a smaller proportion are placed on the waiting list in a timely manner.^1^ The process of being evaluated as a candidate for the waiting list involves multiple steps and transitions of care between the referring nephrologist and the transplant center. During the evaluation process, patients are required to meet with clinical and financial coordinators, social workers, and physicians within the transplant center.^2^ These visits occur in a single day, resulting in deluge of information for patients who also often leave with additional requirements for testing, appointments, and vaccinations. This occurs without adequate input from the dialysis team who know the patient well or have important clinical insights. Patients may be unsure about where they are in the multistep evaluation process, their waiting list status, and the nuances of the impact of consenting for different donor types.^3^^,^^4^

The probability of receiving a deceased donor transplant after wait-listing is contingent upon maintaining an active status and varies based on whether patients have opted to receive offers for organs that require special consent, such as those from donors with hepatitis C virus (HCV) or high kidney donor profile index (KDPI) and considered potential living donors.^5^, ^6^, ^7^, ^8^ More than 45% of kidney transplant candidates are inactive on the kidney transplant waitlist, thus precluding them from being eligible to receive offers.^9^ Nationally, about 2 in 5 waitlisted candidates have opted in for high-KDPI offers with a similar proportion opting-in for HCV-positive donor offers. There is compelling evidence that transplantation using these organs provides better survival and quality of life benefits compared to remaining on dialysis, especially for candidates with longer expected wait times.^10^

Leveraging longitudinal care relationships and empowering patients and dialysis teams to have conversations about waiting list status and types of deceased donor organ offers may improve rates of kidney transplantation, shorten waiting times, and confer a survival benefit compared to remaining on dialysis.^11^^,^^12^ Dialysis teams have more opportunity for engagement with patients compared to transplant teams, especially in the pretransplant period of care. The purpose of this study was to develop and refine patient-centered communication tools for shared decision-making conversations about maintaining active status, living donation, and consent for HCV and high-KDPI organ offers.

## Methods

### Theoretical Framework

We engaged the AHRQ-SHARE model to guide the study design.^13^ The SHARE model is a 5-step process for shared decision-making that creates a dialogue with patients in a manner that helps them make decisions and includes an assessment of the benefits and risks of options viewed through the lens of a patient’s own values and preferences. Using the SHARE approach, we specifically sought to develop a communication tool that would be used to (1) seek waitlisted patients to engage in dialogue with their dialysis teams, (2) help patients to understand the nuances of their status on the waiting list and specific types of organ offers to potentially shorten waiting time and improve rates of transplantation, and (3) assess the patient’s preferences. Ultimately, the patient can then be better positioned to reach a decision and evaluate it with the transplant team. The communication tools provide data from transplant centers to promote dialogue within dialysis facilities among patients, providers, and peers.

### Study Design

We performed a qualitative study using virtual focus groups to engage dialysis and transplant providers, patients, and care partners. Purposive sampling was used to recruit focus group participants in collaboration with the National Kidney Foundation (NKF) advocacy team. Because kidney transplant involves multidisciplinary teams across different organizations including dialysis teams and transplant centers, we specifically sought to include providers as participants from varying disciplines (eg, nurses, social workers, dietitian , and dialysis technicians), patients in various stages of kidney disease, and overall representation from a diverse geographic region. The NKF advocacy team sent email invitations to potential participants who are diverse advocates for kidney health based on their personal and professional experiences. Respondents were subsequently contacted by the NKF team to form each of the focus groups at mutually agreed dates and times. Focus groups were held virtually using a cybersecure platform. *A priori*, focus groups were organized by affiliation to kidney disease; exclusively patient or provider groups held separately. Care partners participated in the patient focus groups. Two rounds of focus groups were undertaken iteratively, with the first aimed at understanding practice patterns and preferences related to shared decision-making and education about transplantation within dialysis facilities. The second round of patient and provider focus groups aimed to evaluate communication tools that were developed by the study team using input from the first round. Participants were asked to review drafts of the communication tool before and during the second round of focus groups. Each focus group lasted ∼60 minutes and had 9 to 12 participants. Four patients and 8 providers participated in both rounds of the focus groups while the balance of each focus group was enriched with new participants. Written informed consent was obtained from each of the participants and a $50 research stipend was provided as compensation for their time. The study was approved by the institutional review boards at Columbia University (IRB-AAAU1202) and Cleveland clinic (IRB- 22-689).

### Research Team

The focus group moderator (AH) was experienced in qualitative research including prior experience in conducting focus groups and did not have a pre-existing relationship with study participants. Each focus group was also facilitated by a member of the NKF advocacy team.

### Data Collection

The research team developed an interview guide, based upon clinical expertise, that the moderator used to guide discussion during the focus groups (File S1). Each focus group was audio recorded and transcribed verbatim by a professional service. Self-reported demographic data was collected from participants before each of the focus groups.

### Communication Tools

Each communication tool was developed using a participatory iterative design process. In the first round of focus groups, we explicitly asked participants to describe the content to include in education materials for dialysis care teams to use based on the context of current practice patterns. The study team decided *a priori* on a communication tool in the form of a letter that was adapted through input from focus groups for each of 4 relevant topics, including: active status, living donation, HCV, and high-KDPI organ offers. Using findings from the first round of focus groups, the study team drafted communication tools on active status and HCV offers. In the second round of focus groups, we specifically asked participants for their impressions on the communication tool drafts, focusing on language, appropriateness of messages, tone, organization, content, statistics, and cultural sensitivity. To objectively assess the content of a subset of the communication tools (wait list status and HCV), participants were asked to complete a modified version of the suitability assessment of materials (SAM) at the start of the second round of focus groups. The SAM instrument was modified to focus on purpose, content, reading level, vocabulary, graphics, captions, layout, and cultural appropriateness of the communication tools.^14^ The remaining two communication tools (living donor and high KDPI) were created to include similar statistics, language, and visuals as the feedback received from the focus groups and SAM instrument for active status and HCV offers.

### Analysis

Descriptive statistics were used to report the characteristics of focus group participants. For thematic analysis, 2 co-authors (AH and SH) independently reviewed all the focus group transcripts using content analysis to create a preliminary code book.^15^ Consensus coding was used to settle discrepancies in the preliminary code book during a dedicated cross-coding session. The 4 focus group transcripts were then re-coded in their entirety applying the final coding scheme. Codes were combined into subthemes and themes to convey our findings.^16^ Distinct themes were identified for patient and provider focus groups. Our operational definition of saturation was pragmatic saturation, or, when the coders found that intended concepts were fully described including: current practices about sharing transplantation information at dialysis facilities and content of a communication tool (first round of focus groups) and the impression on specific categories of the content of draft communication tool (second round of focus groups) from the individual transcripts.^17^

The total suitability score for the content of the communication tool was calculated from the responses on the SAM instrument. The scoring rubric assigned 2 points for superior rating, 1 point for adequate rating, 0 for not suitable ratings. The total SAM score is reported as a percentage of total possible points and was evaluated for deficiencies, or unsuitable ratings, for each of the factors.

The consolidated criteria for reporting qualitative research checklist was used as framework to report on the research team, study design, and analysis.^18^ (File S2)

## Results

In total, 28 stakeholders participated in 4 separate focus groups (2 patient and 2 provider). Participants’ mean age was 49 years; 61% were female, 43% self-reported as White, 14% as Black, and 43% as other races. Most patient participants had a bachelor’s degree (53%), while providers were most likely to have a master’s or doctorate degree (69%). A small number (13%) of the patient participants were care partners, and a majority were otherwise connected to kidney disease through transplant (60%). On average, providers worked at their jobs for 18 years. Among the 13 providers, 4 (30%) were physicians, 3 (23%) were social workers, and the rest were evenly represented by 2 registered nurses, 2 advanced practice providers, and 2 dialysis technicians (15% each). (Table 1)

**Table 1:** Characteristics of Focus Group Participants (n = 28)

Characteristics	Patient (n = 15)	Provider (n = 13)
**Age, mean ± SD**		
Y	46 ± 14	54 ± 14
**Gender, n (%)**		
Male	7 (47%)	4 (31%)
**Race, n (%)**		
White	8 (53%)	4 (31%)
Black	2 (13%)	2 (15%)
Hispanic	3 (20%)	2 (15%)
Asian	0 (0%)	4 (30%)
American Indian/Alaskan Native	2 (13%)	0 (0%)
Native Hawaiian/Pacific Island	0 (0%)	1 (8%)
Mixed	1 (7%)	0 (0%)
**Highest level of education, n (%)**		
High school	2 (13%)	0 (0%)
Some college	3 (20%)	3 (23%)
Bachelors	8 (53%)	1 (8%)
Masters	1 (7%)	5 (38%)
Doctoral	1 (7%)	4 (31%)
**Connection to kidney disease, n (%)**		
Patient with CKD	2 (13%)	
Dialysis	1 (7%)	
Transplant candidate/recipient	9 (60%)	
Both dialysis and transplant	1 (7%)	
Care partner	2 (13%)	
**Discipline, n (%)**		
Dialysis technician		2 (15%)
Registered nurse		2 (15%)
Social worker		3 (23%)
Advanced practice provider		2 (15%)
Physician		4 (30%)
**Years at job, mean ± SD**		18 ± 13

Abbreviations: CKD, chronic kidney disease; SD, standard deviation.

We identified salient themes and subthemes from the focus groups. In the first round of focus groups, the main themes from provider focus groups emerged as current practice patterns, factors that acted as barriers and facilitators to transplant discussions, as well as key considerations for designing a communication tool. Main themes that emerged from the first round of provider and patient focus groups including education and shared decision-making experiences, knowledge and knowledge-seeking behavior, and characteristics of a patient-centered communication tool. Both groups found peer support and modeling valuable, stressed the importance of understanding the evaluation and wait-listing process, as well as getting this knowledge to patients in a way that they can understand. Among these main themes, several subthemes also emerged that varied between providers and patients.

Provider Themes: Shared Decision-Making.

### Practice Patterns

Current practice patterns were described as delivering cursory information about transplantation. Information content was largely transactional and entailed giving transplant center contact information and access to websites, communicating with transplant centers about referrals and deficiencies in evaluations for patients already referred, and checking in with patients to see that they are making progress with their evaluations. (Table 2, Theme A)

**Table 2:** Selected Illustrative Quotes About Shared Decision-Making

Providers		
Theme	Subtheme	Illustrative Quote (Role)
(A) Practice patterns	Communication between transplant center and patients	“We have five transplant hospitals in [our area] and I give them some information on each, the applications or separate the process of the evaluation with them going through the process is also a process of them learning about what transplant is more… I work for a large practice of kidney doctors and then also refer to some websites. And if they ask for statistics, then I'll guide them through the SRTR website” -CKD clinic social worker
		“We just have them call the transplant center periodically just to find out where they are on the list and that helps. When they can hear that they're moving up, I think it gives them a lot of encouragement. And when they get to page two, then they know that they're going to get called a little more often or called for backup. So that helps. We just have them do that two or three times a year.” -Dialysis nurse
	Reinforce ideas of transplant and check-in with patients	“I know that with patients, …you have to really stay on top of them to educate them because many times we refer them to a transplant center and then they automatically assume their first visit they're on the list… If they move, if they change phone numbers, whatever, not only did they let the clinic know, but they have to let the transplant center know…They have to stay on top of it…I mean the dialysis social worker to do everything for them, they don't move those patients quickly through the transplant process because it doesn't show that they're proactive…[social workers] have to stay on top of the patient as far as, where are you in transplant? When was the last time you called?” -Dialysis social work manager
(B) Barriers to shared decision-making conversations	Frontline staff have knowledge gaps	“I think as technicians, we're very ill-equipped to actually deal or encourage patients as to the processes and what needs to be done. Your frontline staff, the technicians are somewhat very uneducated as to how to help patients actually be on the transplant list or encourage them to be on transplant lists, except for us telling them stories about successful transplants.” -Dialysis technician
	Complacency	“A lot of them is just like, ‘Oh, I've seen them [transplant center]. I've met with them. I don't know what's next.’ … And of course we all, based on the knowledge that the waiting list is long, so people just kind of like, ‘Oh, I'm going just have to wait.’ And then because of this anticipation or this assumption that we're just waiting, they wouldn't proactively, very few of them proactively go and call and say, ‘What is my status?’ -Dialysis nurse
	Health literacy	“However, some of our lower socioeconomic patients in my region…read at the third to fifth grade education level and I found that to be true. So I think some of the pamphlets that are short, concise, easily read, and with pictures will be the best thing for them to have as an adjunct to talking to someone like the social worker, the nephrologist, or the team.” -Dialysis technician
(C) Facilitators to shared decision-making conversations	Peer modeling	“I think patient success stories can be very empowering and impactful…I had a patient who took a hepatitis C kidney, took the medication, cured the hepatitis C, because she was in her seventies…She's done well for several years, and has not come back to dialysis. I think patient success stories, and how their thought processes have gone through it and made some decisions, might be impactful.” -Dialysis social worker
		“…we usually encourage patients that have gone to transplant to come back and visit the facility and talk to a couple patients that have been identified that they are higher up on the list to share their experiences good and bad…” -Dialysis social worker
	Motivated patients	“I can tell when they come to the clinic, usually those who are proactive, they get kidneys. It's simple. But the ones who are not, they lag, they lag, and then they think they're still on the list or they think they're on the list. And we do have some people who they are very proactive, they get on about four or five different lists. They don't stay just on one list. They're told by somebody who's interested, and they get on two different ones in Georgia, the one in Alabama, New York. They get all over. So it just depends upon the patient, I believe.” -Dialysis technician
	Physicians set expectation for kidney offer outcomes	“… it has to be spelled out clearly over many visits, a lot of questions answered because I don't want the patient, and I think some of them do feel like they're given a shoddy kidney, and that someone's just trying to dump a kidney on them and told, ‘Oh, this is your best choice.’ So at the end of the day, they need to feel comfortable. They need to have all the information. And I hope that the transplant team will also give them advice. What are their own comorbidities, life expectancy versus getting a kidney that's not the best kidney out there, the risks and benefits. So I hope that's all really spelled out early on.” -General nephrologist
(D) Design of communication tool	Individualized	“…having all levels of staff talk to them because some conversations resonate more than others, but then also making sure that the conversations they are receiving are kind of coordinated…” -Dialysis social worker
	Attention to health literacy and multimodality	“I think some of the pamphlets that are short, concise, easily read, and with pictures will be the best thing for them to have as an adjunct to talking to someone like the social worker, the nephrologist, or the team concept.” -Dialysis technician “We always gear our education materials to a third grade level. And like you said, pictures are worth a million dollars. Keep it simple.” -Dialysis social worker
	Training for dialysis staff to have a basic understanding of transplant	“We have monthly classes with the social workers, because as you're right, we are the frontline people. We don't know all the facts, but we know enough to get their paperwork done in time, to ask them those pertinent questions, to make sure their lab work's being drawn, to make sure they're following up with those other appointments that they have to have…” -Dialysis technician
**Patients**		
**Theme**	**Subtheme**	**Illustrative Quote**
(E) Patient experiences with transplant conversations	Education and practice patterns limited to dialysis-centric information	“When you're in the clinic, even when they're giving education, it all centers around just doing dialysis, not anything post dialysis or anything to do with getting off dialysis. It all centers around how to eat while you're on dialysis, making sure that you come to every treatment. That's it.” -Transplant recipient
	Value of peer support	“That's one of the best ways to teach patients about their disease is with another patient who has the disease, who's gone through the process, knows it firsthand, knows the emotions, knows the lingo, can talk high, can talk our clinical, those things, and can connect on a one-on one.” -Patient with CKD
(F) Knowledge and knowledge-seeking behavior	Knowledge about extensive evaluation	“I wasn't really aware of what was going to be involved with it until I was told to start the evaluation process. It was a full day at the hospital going from one location to another location. The blood tests alone with 22 vials, and it took an hour and a half just to pull that, and then there was more later on. Chest x-rays, EKG, and then I had to go back and have nuclear tests. It was quite extensive… If I knew up front what I was going to go through, I would've been a lot more comfortable.” -Patient with CKD
	Perceived risks, benefits and outcomes depends on severity of illness	“…because I'm in a situation where I'm not currently on dialysis and not at the point where I'm going to need to immediately start dialysis right away at least, I would not be willing. I'd be very reluctant to have a kidney from someone that had hepatitis or that sort of thing. But I understand that for other people in a worse situation, they may feel they have really nothing to lose and I'm not there yet.” -Patient with CKD
		“I think that you have to frame it in terms of how bad do you want freedom? I think that a lot of folks who have been on dialysis, myself included, we're tired. I mean, we're just tired. I think that probably at the end of the day, we're exhausted. We deal with a lot of things every other day on dialysis, and this is just one more thing that I have to go through, one more thing I have to deal with.” -Transplant recipient
(G) Characteristics of patient-centered communication tool	Individualized to level of health literacy	“You would need to evaluate the person and their capability of understanding, their level of education. That right there is detrimental. That is the first step. You have to understand their level of understanding, and then you would have to get the material to fit that person because everyone's level of understanding and level of education is not the same. That's one. That is a big impediment to education in the clinic.” -Transplant recipient
	Multiple formats	“…you have to come up with different ways of hitting people. Some people read, some people listen, some people have to do. So you have to have multiple resources of materials. And I'm not in dialysis, but I would think that would be a very difficult time as it is in itself to try and learn new material.” -Patient with CKD
	Present all options	“I think to introduce different modalities of transplantation, whether it be, ‘Hey, you can receive a transplant from a Hep B, Hep C individual, or if you have HIV/AIDS, you can receive another transplant from someone with HIV/AIDS.’ Something to that effect, to where you have this plethora of options that you're not pigeonholed per se into just thinking, ‘Okay, I need to get on a list. I need a transplant, but it's going to take me seven years or whatever it may be.’ That there has to be education to coincide with the fact that there are a lot of different options out there as far as receiving a transplant.” -Transplant recipient

Abbreviations: CKD, chronic kidney disease; EKG, electrocardiogram; SRTR, Scientific Registry of Transplant Recipients.

### Barriers and Facilitators

Providers eloquently described multiple barriers to conversations and shared decision-making during dialysis sessions. Patients often have difficulty accepting the recommendations and advice from providers due to fear, preconceived notions, and witnessing other patients who experience adverse transplant outcomes. Even though frontline dialysis personnel build trusting relationships with patients, they reported, “being somewhat [or] very uneducated as to how to help patients be on the transplant list or encourage them to be on the transplant lists, except for…telling them stories about successful transplants.” This superficial knowledge about the nuances of the kidney transplant process was tempered by anecdotal firsthand experience. Providers inferred that many patients are complacent as they feel well during dialysis and “very few of them proactively go and call and say, What is my status?” to engage in conversations with their transplant teams. Moreover, low health literacy contributes to patients’ understanding of written materials. (Table 2, Theme B)

Connecting with other patients who have received kidney transplants within the dialysis unit, patients who are proactive and self-motivated to complete the multi-step process were identified as facilitators to discussions about transplantation. Physicians engaging with patients transparently to set expectations about the types and quality of kidneys that may benefit a certain patient early in the transplant process was also identified as a facilitator to shared decision-making. (Table 2, Theme C)

### Design of Communication Tool

Providers emphasized that the communication tool should be individualized, tailored to the appropriate level of health literacy, and delivered within the dialysis facility using multiple modalities. Additionally, providers indicated that dialysis staff should be trained to have a basic understanding of transplant to deliver a unified message to patients and so that they can dispel misconceptions about transplant by providing accurate information. (Table 2, Theme D)

### Patient Themes: Shared Decision-Making

#### Experiences

Patients described limited engagement in shared decision-making discussions with their dialysis teams, noting that interactions narrowly focused on dialysis-specific topics. Patients largely entrusted advice from “another patient who’s gone through the process, knows it firsthand, knows the emotions, knows the lingo…and can connect on a one-on-one” basis over advice from dialysis team members. (Table 2, Theme E)

#### Knowledge and Knowledge-Seeking Behavior

Complexities of the kidney transplant evaluation process and nuances about organ offer types are poorly understood by dialysis patients who explained, “that there's an awful lot of confusion and an awful lot of, I won't say necessarily misinformation, but it's not very clear how the listing process tends to work for patients…or…for families to understand.” The multiple steps involved in an initial transplant evaluation are not transparent until patients are during the process. Moreover, knowledge-seeking behavior about different types of deceased donor organs and strategies to minimize waiting time is closely associated with severity of dialysis-associated symptoms and overall comorbidity. As a result, the perceived risks, benefits, and outcomes of riskier organ offers may change over time. (Table 2, Theme F)

#### Characteristics of Patient-Centered Communication Tool

Like providers, patients also agreed that communication tools should be individualized to meet their needs, including written at the appropriate level of health literacy. Also, patients highlighted the need for the communication to occur in multiple formats. Patients felt that all types of donor options should be presented, including explicitly describing the anticipated post-transplant course for HCV-positive and high-KDPI organ offers. (Table 2, Theme G)

### Provider and Patient Themes: Communication Tool

**Figure 1:**
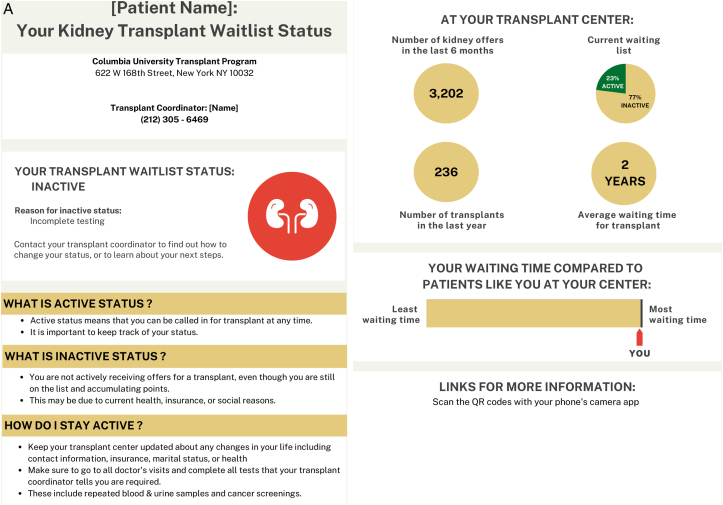

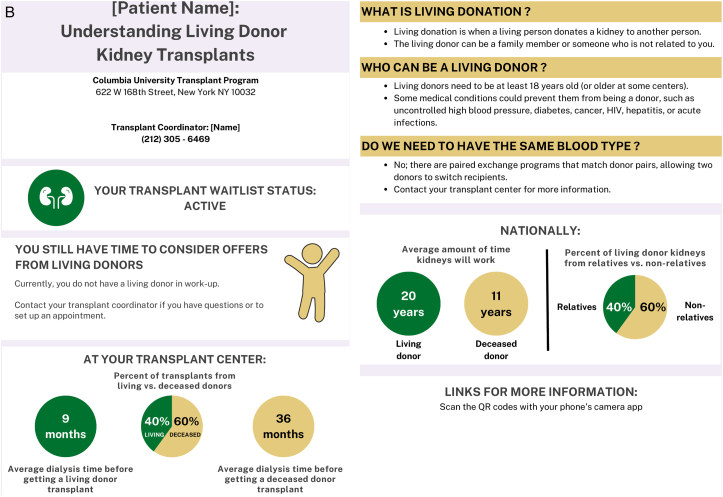

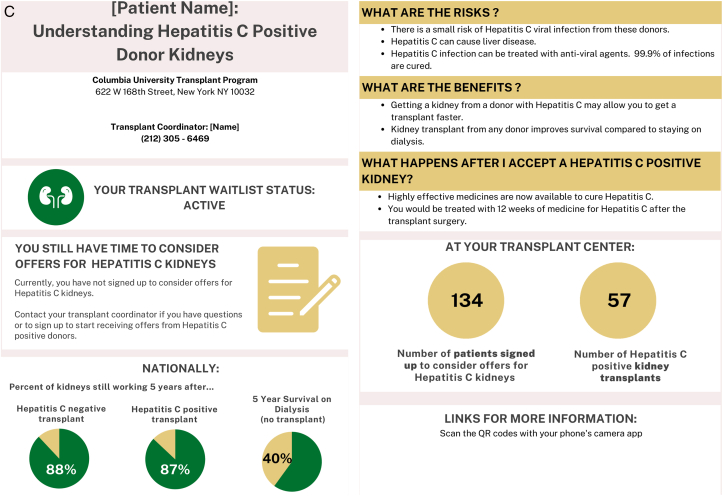

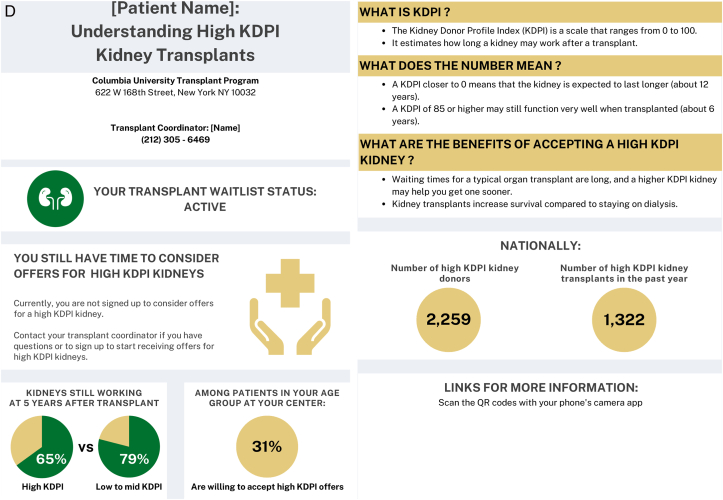
Suitability assessment measurement scores of communication tools.

In the second round of focus groups, themes and subthemes emerged around provider and patient impressions of the proposed communication tools. (Fig 1A-D) Providers and patients both shared overall positive and negative impressions and commented specifically on the layout and content of the communication tools. Themes around implementation of the communication tools also emerged.

Providers found that the messaging of the communication tool was most impactful with statistical information, which “brings a reality to how much work is actually being done through the comprehensive team.” However, patients tended to favor content that included benefits and outcomes of the specific donor types as well as the next steps. (Table 3, Theme A) The main opportunity for simplification of the communication tool was to eliminate the use of a quick response (QR) code as part of the layout. Both patients and providers felt it added unnecessary complexity to the communication tool and not an ideal way to provide access to additional information. (Table 3, Theme B)

**Table 3:** Selected Illustrative Quotes From Patients and Providers About the Communication Tool

Theme	Subtheme	Illustrative Quote (Role)
(A) Content	Objective information helpful	“…I will say that I really like…the second section that gives the stats across the transplant center. I think patients normally don’t hear how successful a transplant center is. And, I think that kind of brings a reality to how much work is actually being done through the comprehensive team.” -General nephrology nurse practitioner “I thought that it was very impactful, especially the 85, 80, and 50 part on the hepatitis C [tool] where it has percentages…we want the patients to infer that they will live longer with a transplant whether it’s hepatitis C or not.” -CKD clinic social worker
	Focus on benefits, outcomes, and next steps	“What are the risks? What are the benefits? What happens after I accept an HCV+ kidney?…or, you need to have somewhere [the patient] can go read more about it.…where they can just go and understand [more].” - Transplant recipient “…the one thing that stands out to me that perhaps might not be so evident is that I’m not sure what I need to do as a recipient once I receive this [communication]. It’s given me great indication of where I stand. It’s just not coming across clearly to me about what I need to do.” - Transplant recipient
(B) Layout	Opportunities to simplify	“I love the idea of the QR codes, but I know the majority of my patients wouldn’t know what to do with that…” -General nephrology physician assistant
(C) Implementation	Enhance usual practice of dialysis team members	“I can tell you that in a normal day to day operation in an outpatient unit, technicians are your most abundant staffing Engaging them to do anything outside of their normal priming, put on and takeoff, is so difficult that if you can come up with that magic bullet it would be perfect.” -Dialysis technician “As a social worker, I would love to do this. I’ve done this with patients just by hearing staff from [transplant center name] come and talk to us about hepatitis C kidneys. Went back and told some patients it was an opportunity for them to consider just from [getting] the knowledge. But having this handout would be much better.” -Dialysis social worker
	Tailor to all age groups	“I think you need to come up with three specific methods to address age groups., likely younger…to middle age…to older age group…because each age groups can be more technically inclined or less technically inclined.” - Transplant recipient
	Establish point of contact at transplant center	“A dialysis center is fine as an educational source, but I would not use it to communicate specifics about a particular patient case without having a doctor or transplant nurse available to respond.” -Patient care partner “Is the person at the clinic going to be in direct contact with whoever’s at the transplant center? Because there might be some confusion if you just come and drop this off…” -Transplant recipient

Abbreviations: CKD, chronic kidney disease; HCV, hepatitis C virus.

Asking dialysis teams to complete extra tasks to implement that communication tool into their workflow was identified as a challenge. However, enhancing the usual practice of dialysis teams (eg, a social worker) by integrating a communication tool about kidney transplantation was deemed very feasible. Adapting the communication tool to accommodate the various age groups who receive dialysis and are eligible for transplant was deemed an important part of implementing it. Although the intent is for the communication tool to be implemented within dialysis facilities, patients were hesitant for dialysis facilities “to communicate specifics about a particular patient case without having a doctor or transplant nurse available to respond”. (Table 3, Theme C)

### Suitability Assessment of Materials

**Figure 2:**
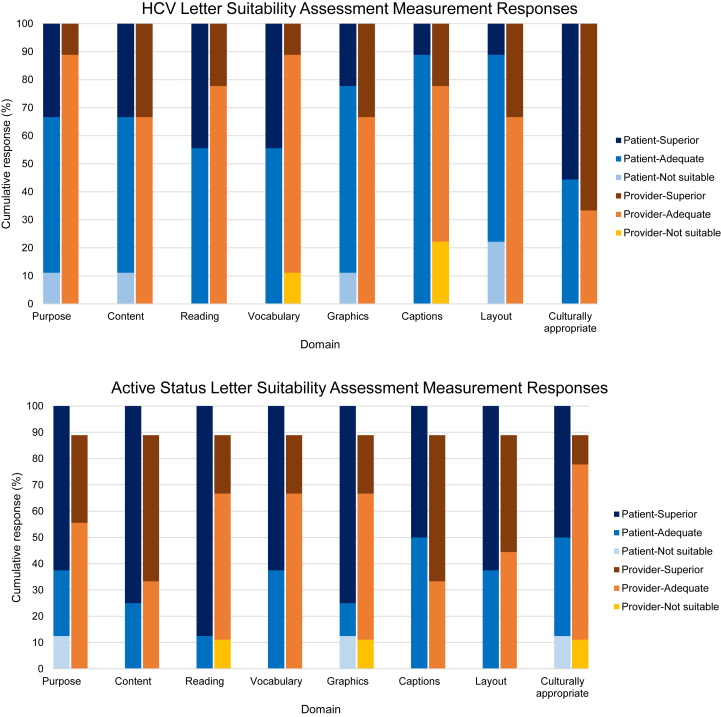
Final communication tools.

Objectively, the SAM scores showed that providers and patients rated the HCV and wait-listing status communication tools overall as superior or adequate in each of the domains. (Fig 2)

The HCV communication tool received a median suitability score of 56.3% (IQR 56.3-68.8) among providers and 62.5% (IQR 50-75) among patients. One provider did not complete any responses on the wait-listing survey and was not included in the scoring for the wait-listing status tool, which received a median suitability score of and 68.8% (56.3-75) among providers and 84.4% (IQR 75-96.9) among patients.

## Discussion

In this qualitative study, we found that dialysis provider discussions are limited, providing only cursory information about kidney transplantation with patients and that communication focuses on dialysis-related issues. Communication and explicitly shared decision-making may be improved with additional provider training, peer mentoring, and expectation-setting by physicians. The content of communication tools should be simple and emphasize the risks, benefits, and expected outcomes of different types of transplants. Additionally, communication tools should be offered in multiple modalities. As a result of our work, letters were developed that will be used in future studies to determine how shared decision-making is impacted around wait listing status and offer types to improve rates of actual kidney transplantation. Determining if the letters promote conversations within dialysis facilities is also important as well as refining the tools to incorporate patient and provider insights as they are tested in future studies. Letters were developed using center- specific metrics from the two centers in this study, they can be modified for use in other US transplant centers by replacing the data elements accordingly.

Although shared decision-making regarding organ offer consent has not been previously studied, our results add to evidence of the value of shared decision-making in other aspects of kidney disease care.^8^^,^^11^^,^^19^, ^20^, ^21^ While patients desire shared decision-making conversations in this context and others, such conversations are often limited or absent.^22^ Previously identified barriers by patients, care partners, and providers include fragmentation of care, lack of collaboration between providers, poor communication styles, patient and provider avoidance, mixed messaging, and physician paternalism.^20^^,^^23^, ^24^, ^25^, ^26^ Because dialysis and transplant are managed in 2 distinct settings and by different care teams, transplant candidates may be uniquely unaware of the best sources of information and counseling to address knowledge barriers related to waitlist outcomes, barriers to transplant, and transplant types. Improving knowledge about transplant referrals, evaluations, and waitlist management for dialysis care teams is a critical component of implementing shared decision making.^27^ With new requirements to document referrals for patients in the first few months of dialysis initiation for patients, the Center for Medicare and Medicaid Services has created a new emphasis on this aspect of patient care.

Decision support tools may improve the frequency and quality of shared decision-making in kidney disease by filling knowledge gaps and stimulating conversations between patients and providers. A prior pilot trial of a decision aid tool about conservative care of kidney disease showed marked increases in discussions about treatment options between patients and both providers and family members.^28^ Our tool, designed based on patient-reported preferences for communication tools and provider-reported knowledge gaps, may similarly increase transplant-related communication between patients, dialysis providers, and transplant centers to ensure that patients’ waitlist and organ offer statuses reflect their priorities and values. The systematic and regular delivery of this tool to patients may also improve equity in transplant by reducing known heterogeneity in transplant-related counseling.^29^, ^30^, ^31^, ^32^ This will require greater commitment from the general nephrology community, including greater awareness of the process that may be achieved through educational requirements of nephrologists and advanced practice providers. Given our finding that patients prefer multiple formats to receive transplant-related material, optimal use of this tool at the dialysis center may be in conjunction with home-delivered transplant interventions, such as one currently being examined in a randomized trial.^33^ Future interventions may also add peer support or counseling components, in light of our findings and others’ that patients view other patients as valuable sources of information and support.^34^

The participants included a demographically and geographically diverse group of patients and providers, including providers from a variety of patient care settings. However, because of the small absolute number of stakeholders in our focus groups, these findings might not represent all practice settings. Since medical decision-making relies on a complex interplay of patient experiences, trust, comprehension of information, and risk-benefit perceptions each patient has unique needs that need to be met to optimize shared decision-making. We acknowledge the limitation that dialysis teams alone may not suffice to help patients make decisions about their transplant preferences from start to finish; however, we showed that dialysis providers can be a valuable resource in the critical first steps of a shared decision-making process. Future work to leverage existing relationships within dialysis care settings is one way to facilitate shared decision-making to account for patient preferences about transplant. However, gaps exist in understanding how to effectively provide information to dialysis teams and seamlessly integrate discussions into dialysis workflows. Future work to further refine patient communication tools and streamline these types of interventions are important next steps.

In understanding current practice patterns and defining facilitators and barriers to shared decision-making among patients and their dialysis providers, we developed a communication tool to be tested in future studies. Opportunities to discuss the risks, benefits, and potential outcomes for different types of donor offers are likely to increase kidney transplantation in a manner consistent with patient preferences.

## Supplementary Material

Supplementary Files 1 (PDFs)

Supplementary Files 2(PDFs)
