# Supplementary material for: Engaging Dialysis Teams in Shared Decision-Making Conversations With Patients to Improve Rates of Kidney Transplantation
Source: Kidney Med. 2026 Feb 27;8(5):101310. doi: 10.1016/j.xkme.2026.101310 (PMC13090513; doi:10.1016/j.xkme.2026.101310)
Supplement: Supplementary Files 1 (PDFs) [file mmc1.pdf]

PROVIDER FOCUS GROUP GUIDE: 1

Hello Everyone. My name is {Facilitator Name}.   I want to thank you for agreeing to be part of this discussion today. As a reminder, the focus group will be recorded, and I will start the recording now.

We're interested in your thoughts and experiences as a provider who is working with patients who are on the kidney transplant waiting list.

There are no right or wrong answers to the questions. In fact, we are doing this research because we think that it is most important for us to understand interactions about kidney transplant that occur between providers and patients within a dialysis facility. Understanding these interactions better will help us to develop patient-centered education materials for dialysis facility staff. The goal of the educational materials will be to initiate conversations about kidney transplants with the staff and clinicians. By engaging the dialysis care team in shared decision-making conversations, patients may more likely remain active on the waitlist, and, think about what organ types they want to consider to improve their chances of getting organ offers and a kidney transplant. Working together, we will use your input to design educational materials and carry out our research study.

Please try to be as honest and open as you can so we can learn from your experiences. However, if there are questions that you do not feel comfortable answering or discussing, you do not have to answer them. Please tell me and we'll move on to the next question. Before we go on, do you have any questions?

PRACTICE PATTERNS

Can you walk me through your current role in assisting or advising patients regarding kidney transplant? Examples of things I would like to know about are:

- Referral and evaluation process
- Testing and consultations that are part of the transplant evaluation
- Promotion of adherence
- Communication with the transplant center
- Addressing life and social concerns
- Education
- Self-management support
- Costs, insurance, finances, or difficulty in getting care or medications
- Difficulty in getting to medical appointments
- Anything else?

What kinds of standard things do you do to help patients who are on the kidney transplant waiting list make decisions?
Examples of things I would like to know about are:

- Education
- Self-management support
- Financial assistance, appointment reminders, arrange transportation
- Communicating with transplant center
- Referrals (support groups, social work, case management)

- Other patient engagement (i.e. Individualized treatment plans, seminars, engage family members)
- Anything else?

In your experience, what kinds of difficulties do patients face in understanding and accepting your recommendations or advice?

- About their kidney failure and treatments in general
- About kidney transplant

How many patients who are on the kidney transplant waiting list would you say that you see in a given week? How often do you discuss kidney transplant with these patients?

BARRIERS AND FACILITATORS

Now I'd like to have a discussion of things that are easiest and most challenging in starting conversations and making mutual decisions about waiting list status and opting in for different organ offers in your patients. The different organ types that we are specifically thinking about are high kidney donor profile index (KDPI) organs, and organs from donors who are positive for hepatitis C.

Working together, we will pair you and another person up in a breakout room. I'd like each pair to make a list of the top 5 things that are easiest and the top 5 things that are most challenging in communicating with patients about decision-making for kidney transplant including maintaining active status on the waiting list, and thinking about which organ types to consent for. We will then go around and share what we've come up with.

[PUT INTO BREAKOUT ROOMS, GIVE PAIRS 2-3 MINUTES TO DISCUSS WITH EACH OTHER] [CONDUCT ROUND ROBIN, ASKING EACH PAIR TO SHARE SOME OF THE THINGS THEY HAVE WRITTEN DOWN, USE CHAT OR OTHER MEANS TO RECORD RESPONSES]

Potential examples of topics that pairs might list:

- Patient knowledge level
- Provider knowledge level
- Communication with transplant team
- Lack of information about transplant outcomes

This next activity is similar. I want you to work together again and make a list of the top 5 things that would help you to improve the education and support that you provide to patients about staying active on the kidney transplant waiting list and different types of organs that they may consider to increase chances at kidney transplant. These don't have to be things you are actually doing now, but they can be. We will then go around and share what we've come up with.

[WAIT 2-3 MINUTES THEN CONDUCT ROUND ROBIN, ASKING EACH PAIR TO SHARE SOME OF THE THINGS THEY HAVE WRITTEN DOWN, USE FLIPCHART TO RECORD RESPONSES]

Potential examples of topics that duos might list:

- Financial assistance
- Patient has support network (family, friends, church, social groups)
- Availability of nurse/physician to answer questions
- Availability of easy-to-understand educational materials
- Better communication between patient/provider or physicians/nurses
- Transportation assistance to get to healthcare
- Professional training

SUGGESTIONS FOR EDUCATIONAL MATERIALS

We want to develop educational and informational material tailored to patients and dialysis care team to empower staff to participate in shared decision making about kidney transplants from different types of organ donors.

Thinking about all patients on the kidney transplant waiting list that you have seen, what types of information seems to help most with their knowledge about waiting list status and likelihood of getting a kidney transplant?

In general, what can be done to better prepare patients for making decisions about organ offers including consenting for high KDPI and hepatitis C kidneys?

More specifically, what format and content should educational materials about waiting listing status and different types of organ offers be made?

How should the educational materials look?

How often should the educational material be reviewed? And, for what duration in each session?

PROVIDER KNOWLEDGE

I want to conclude the focus group by asking what your own comfort level is in understanding around the topics that we discussed today.

Under what circumstances do you think patients should or should not consent for high KDPI and hepatitis C organs?

Explain your comfort level or limitations in:

Educating patients about what KDPI means?

Educating patients about hepatits C transplantation?

Is there anything else that we have not covered that you feel is important?

Thank you very much for joining in the discussion. I appreciate your time in talking with us today and the ideas you have shared will be instrumental in promoting shared decision making about organ transplant offers and wait listing status.

PATIENT/CARE PARTNER FOCUS GROUP GUIDE:1

Hello Everyone. My name is {Facilitator Name}. I want to thank you for agreeing to be part of this discussion today. As a reminder, the focus group will be recorded, and I will start the recording now.

We're interested in your thoughts and experiences as a patient with kidney disease or a caregiver for someone with kidney disease.

We are doing this research because we think that it is most important for us to understand interactions about kidney transplant that occur between healthcare providers and patients within a dialysis facility. Understanding these interactions better will help us to develop patient-centered education materials for dialysis facility staff. The goal of the educational materials will be to initiate conversations about kidney transplant between patients, dialysis staff, and clinicians. By engaging in the dialysis care team in shared decision-making conversations, patients may more likely remain active on the waitlist, and, think about what organ types they want to consider to improve their chances of getting organ offers and a kidney transplant. Working together, we will use your input to design educational materials and carry out our research study.

Please try to be as honest and open as you can so we can learn from your experiences. There are no right or wrong answers to the questions that we discuss today. However, if there are questions that you do not feel comfortable answering or discussing, you do not have to answer them. Please tell me and we'll move on to the next question. Before we get started, does anyone have questions?

Wait Listing and Organ Offer Knowledge

To start off, I would like to hear what the group knows about the kidney transplant waiting list?

If someone asked you what the kidney transplant waiting list is, what is/are something(s) you think would be important for them to know?

If someone asked you about kidney transplants from organ donors that may have had health problems, or who may have had hepatitis C, what would you tell them?

We know that accepting high kidney donor profile index (KDPI) organ offers or organs from donors with hepatits C infection helps patients live longer since the benefit of getting a transplant faster outweighs waiting longer for other kidneys. But most patients do not consent to receive these offers, why do you think that is the case?

Experiences

Can a few of you please walk me through your decision to pursue kidney transplant?

Can a few of you please walk me through how the kidney transplant wait listing process went for you?

Could you tell me about things that made the transplant work-up and wait listing process for you easier or harder?

For those of you who received a kidney transplant, tell me what you remember about conversations with your transplant team about different types of kidney donors?

What about conversations with your dialysis care team about different types of kidney donors?

Thinking back, or, from what you know now, how would you explain KDPI to another patient?

Thinking back, or, from what you know now, how would you explain receiving a transplant from a donor with hepatitis C?

How did your dialysis care team help you to understand your transplant options; especially while you were undergoing dialysis and waiting for a kidney transplant?

What types of things could your dialysis care team have done to help more?

For those of you thinking about a kidney transplant, let's pretend that you are trying to get onto the transplant waiting list. What things can your dialysis care team do to help you?

How confident do/did you feel about deciding to agree to a kidney organ offer when you are/were called from the transplant center?

Tell me about how you make decisions regarding your health. Who do you trust? What do you find helpful?

How do you make sure that your own values and beliefs are considered when making decisions about your healthcare?

Educational Materials

What types of things are helpful for you to learn about your kidney disease and transplant?

What are some challenges you have faced in learning about your kidney disease and transplant?

Imagine you were asked to help create educational materials for your kidney/dialysis care team to help new patients understand their waiting listing status and how organ offers occur. What would the materials look like?

What things would you make sure were part of the materials?

What things would you NOT include in those materials?

Is there anything else that we have not covered that you feel is important for us to know?

Thank you very much for joining in the discussion. I appreciate your time in talking with us today and the ideas you have shared will be instrumental in promoting shared decision making about organ transplant offers and waiting listing status.

PROVIDER FOCUS GROUP GUIDE: 2

Hello Everyone. My name is {facilitator name} and I'm a {title} at {hospital system affiliation}. I want to thank you for agreeing to be part of this discussion today. We know that you are all busy professionals and sincerely appreciate your time and expertise as participants. Also, thanks to any of you who also participated in the first focus group.

Before we get started, I also wanted to introduce the other research study team members on the meeting today.

A few quick reminders about using Zoom:

-if you are in a place with background noise, please mute your microphone when not talking

-if you are comfortable leaving your video on, that would be great so that we can keep things conversational

-X and Y will help me to monitor the chat section and hand raising during the focus group today, so feel free to use these options to get our attention. Otherwise, just feel free to speak up during the discussion.

As a reminder, the focus group will be recorded, and I will start the recording now.

We are interested in your thoughts and experiences as a provider who is working with patients undergoing dialysis who are also on the transplant waiting list.

There are no right or wrong answers to the questions during today's focus group. In fact, we are doing this research because we want your feedback on patient-centered education materials for dialysis facility staff. Our goal is to develop educational material tailored to patients and their dialysis care teams to empower staff to participate in shared decision making about kidney transplants from different types of organ donors.

In our last round of focus groups, we sought initial opinions about practice patterns, facilitators and barriers to conversations about transplant, provider knowledge, and educational materials.

We now seek your opinions about the drafts of patient-facing materials that will be used to facilitate shared decision-making about: *Hepatitis C Positive Donor Kidneys and Kidney Transplant Waitlist Status*.

Working together, we will use your input to revise and edit our educational materials and carry out our research study.

Please try to be as honest and open as you can. However, if there are questions that you do not feel comfortable answering or discussing, you do not have to answer them. Please tell me and we will move on to the next question. Before we go on, do you have any questions?

Overall Impressions

Can you please share your first impressions of the **kidney transplant waitlist status patient letter**? [not everyone needs to comment as there will be a lot of opportunities to give feedback on the letters]

Next, can you please share your first impressions of the **hepatitis C positive donor kidneys patient letter**?

Next, I would like to have half of the participants focus on the **kidney transplant waitlist status patient letter**. (Identify names for half of the group) In a round robin fashion, please state 1-2 things that you like the most and 1-2 things that you like least on the letter.

Now, I would like to do the same with the other have of the participants focusing on the **hepatitis C positive donor kidneys patient letter**. (Identify names for half of the group) In a round robin fashion, please state 1-2 things that you like the most and 1-2 things that you like least on the letter.

<u>Impressions on Content</u>

For the next set of questions, I want to find out your impressions about the content of the patient letters. Let's begin by focusing on the **kidney transplant waitlist status patient letter**.

What are your impressions about the kinds of information displayed in the letter? Can you please comment specifically on:

- Language used? (understandable)
- Appropriateness of the messages?
- Tone? (positive and respectful)

What are your impressions about the general layout?

Can you please comment specifically on:

- Organization? (readability)
- Font size?

What are your impressions on the content?

Can you please specifically comment on:

- Statistics?
- Questions and answers?
- Culturally sensitive and non-offensive?

Next, let's focus on the **hepatitis C donor kidneys patient letter**.

What are your impressions about the kinds of information displayed in the letter? Can you please comment specifically on:

- Language used? (understandable)

- Appropriateness of the messages?
- Tone? (positive and respectful)

What are your impressions about the general layout?

Can you please comment specifically on:

- Organization? (readability)
- Font size?

What are your impressions on the content?

Can you please specifically comment on:

- Statistics?
- Questions and answers?
- Culturally sensitive and non-offensive?

<u>Letter Implementation</u>

Imagine that you are asked to use these patient letters in dialysis facilities to improve communication about kidney transplant to help patients get waitlisted, maintain active status on the waitlist, and consider which organ types they want to consider improving their probability of transplantation in a manner that is consistent with their preferences.

What types of things may make it harder or easier to use these letters to improve communication and shared decision-making between dialysis providers and patients?

When is it best to start these conversations?

What training about the letters will dialysis providers need?

As a follow-up question, what information may be included on a sample letter to help que dialysis providers about what to say to patients?

How can the study team best support dialysis providers as the letters are distributed and discussions occur?

What format for delivering these letters Is most effective, or, most preferred by patients and why? (e.g. U.S. mail to house, email, etc.)

How do you think dialysis patients will react to these letters?

Is there anything else that we have not touched on today that you want us to know?

Thank you very much for joining in the discussion. I appreciate your time in talking with us today and the ideas you have shared will be instrumental in promoting shared decision making about organ transplant offers and waiting listing status.

PATIENT/CARE PARTNER FOCUS GROUP GUIDE: 2

Hello Everyone. My name is {facilitator name} and I'm a {title} at Cleveland Clinic. I want to thank you for agreeing to be part of this discussion today. We know that you are all busy and sincerely appreciate your time and expertise as participants. Also, thanks to any of you who also participated in the first focus group and have returned for round two.

Before we get started, I also wanted to introduce the other research study team members on the meeting today.

A few quick reminders about using Zoom:

-if you are in a place with background noise, please mute your microphone when not talking

-if you are comfortable leaving your video on, that would be great so that we can keep things conversational

-Jessica and Haley will help me to monitor the chat section and hand raising during the focus group today, so feel free to use these options to get our attention. Otherwise, just feel free to speak-up during the discussion.

As a reminder, the focus group will be recorded, and I will start the recording now.

We are interested in your thoughts and experiences as a patient with kidney disease or the care partner of a patient with kidney disease who may be on the transplant waiting list.

There are no right or wrong answers to the questions during today's focus group. In fact, we are doing this research because we want your feedback on patient-centered education materials for dialysis facility staff to use. Our goal is to develop educational material tailored to patients and their dialysis care teams to empower staff to participate in shared decision making about kidney transplants from different types of organ donors.

In our last round of focus groups, we sought initial opinions about your decision making and designing educational materials.

We now seek your opinions about the drafts of patient-facing materials that will be used to facilitate shared decision-making about: *Hepatitis C Positive Donor Kidneys and Kidney Transplant Waitlist Status*. Each of you should have received a hard copy of the materials that you may want to have handy as a reference during today's discussion.

Working together, we will use your input to revise and edit our educational materials and carry out our research study.

Please try to be as honest and open as you can. However, if there are questions that you do not feel comfortable answering or discussing, you do not have to answer them. Please tell me and we will move on to the next question. Before we go on, do you have any questions?

To start, we will have each of you respond to a quick poll about the specific letter that we are going to talk about. In the first part of the discussions, I will ask about your overall first impressions. Then, we will talk separately about the content and layout of the letter. Finally, we will talk about the feasibility of using these letters in dialysis facilities.

{NKF puts up the SAM questions to poll participant about the **kidney transplant waitlist status patient letter**}

<u>Overall Impressions</u>

Can you please share your first impressions of the **kidney transplant waitlist status patient letter**? [not everyone needs to comment as there will be a lot of opportunities to give feedback on the letters]

Next, I would like to have half of the participants focus on the **kidney transplant waitlist status patient letter**. (Identify names for half of the group) In a round robin fashion, please state 1-2 things that you like the most and 1-2 things that you like least on the letter.

<u>Impressions on Content</u>

For the next set of questions, I want to find out your impressions about the content of the patient letters. Let's begin by focusing on the **kidney transplant waitlist status patient letter**.

What are your impressions about the kinds of information displayed in the letter? Can you please comment specifically on:

- Language used? (understandable)
- Appropriateness of the messages?
- Tone? (positive and respectful)

What are your impressions about the general layout?

Can you please comment specifically on:

- Organization? (readability)
- Font size?

What are your impressions on the content?

Can you please specifically comment on:

- Statistics?
- Questions and answers?
- Culturally sensitive and non-offensive?

In the next part of the discussion, let's turn our focus to the **hepatitis C positive donor kidneys patient letter**.

{NKF puts up the SAM questions to poll participant about the **hepatitis C positive donor kidneys patient letter**}

<u>Overall Impressions</u>

Can you please share your first impressions of the **hepatitis C positive donor kidneys patient letter**?

Now, I would like to do the same as we did earlier with the other half of the participants focusing on the **hepatitis C positive donor kidneys patient letter**. (Identify names for half of the group) In a round robin fashion, please state 1-2 things that you like the most and 1-2 things that you like least on the letter.

<u>Impressions on Content</u>

Let's think about the content that is in the **hepatitis C donor kidneys patient letter**.

What are your impressions about the kinds of information displayed in the letter? Can you please comment specifically on:

- Language used? (understandable)
- Appropriateness of the messages?
- Tone? (positive and respectful)

What are your impressions about the general layout?

Can you please comment specifically on:

- Organization? (readability)
- Font size?

What are your impressions on the content?

Can you please specifically comment on:

- Statistics?
- Questions and answers?
- Culturally sensitive and non-offensive?

Letter Implementation

Imagine that you are being treated in a dialysis facility for your kidney disease and these letters are being used to start conversations about kidney transplant. Specifically, in order to help patients get waitlisted, maintain active status on the waitlist, and consider which organ types they want to consider improving their probability of transplantation in a manner that is consistent with their preferences.

What types of things may make it harder or easier to use these letters to improve communication and shared decision-making between dialysis providers and patients?

When is it best to start these conversations?

How can the study team best support dialysis patients as the letters are distributed and discussions occur?

What format for delivering these letters Is most effective, or, most preferred by patients and why? (e.g. U.S. mail to house, email, etc.)

How do you think you would react to these letters? What would you do if you received it?

Is there anything else that we have not touched on today that you want us to know?

Thank you very much for joining in the discussion. I appreciate your time in talking with us today and the ideas you have shared will be instrumental in promoting shared decision making about organ transplant offers and waiting listing status.
